# Tunneling nanotube (TNT) formation is downregulated by cytarabine and NF-κB inhibition in acute myeloid leukemia (AML)

**DOI:** 10.18632/oncotarget.13853

**Published:** 2016-12-10

**Authors:** Maria Omsland, Øystein Bruserud, Bjørn T Gjertsen, Vibeke Andresen

**Affiliations:** ^1^ Centre for Cancer Biomarkers CCBIO, Department of Clinical Science, Precision Oncology Research Group, University of Bergen, Bergen, Norway; ^2^ Leukaemia Research Group, Department of Clinical Science, University of Bergen, Bergen, Norway; ^3^ Department of Internal Medicine, Haematology Section, Haukeland University Hospital, Bergen, Norway

**Keywords:** acute myeloid leukemia, tunneling nanotubes, cell-to-cell communication, chemotherapeutics, NF-κB

## Abstract

Acute myeloid leukemia (AML) is a bone marrow derived blood cancer where intercellular communication in the leukemic bone marrow participates in disease development, progression and chemoresistance. Tunneling nanotubes (TNTs) are intercellular communication structures involved in transport of cellular contents and pathogens, also demonstrated to play a role in both cell death modulation and chemoresistance. Here we investigated the presence of TNTs by live fluorescent microscopy and identified TNT formation between primary AML cells and in AML cell lines. We found that NF-κB activity was involved in TNT regulation and formation. Cytarabine downregulated TNTs and inhibited NF-κB alone and in combination with daunorubicin, providing additional support for involvement of the NF-κB pathway in TNT formation. Interestingly, daunorubicin was found to localize to lysosomes in TNTs connecting AML cells indicating a novel function of TNTs as drug transporting devices. We conclude that TNT communication could reflect important biological features of AML that may be explored in future therapy development.

## INTRODUCTION

Acute myeloid leukemia (AML) is an aggressive bone marrow derived blood cancer [1–3] where the bone marrow represents a multi-cellular compartment with a high level of cell-to-cell communication [4, 5]. The cellular communication between blood cells and stromal cells may be exploited by the leukemic cells for increased survival and resistance to chemotherapy [6, 7]. Tunneling nanotubes (TNTs) are novel cell-to-cell communication devices [8] demonstrated to connect a variety of cell types including primary cells and cancer cells [9, 10]. TNTs are defined as thin (50-200 nm), F-actin containing plasma membrane embedded tunnel-like structures (5–100 μm) that interconnect cells by hovering above the substratum [8]. TNTs and TNT-like structures have been shown to transport different cellular cargos, including endocytic vesicles, mitochondria, lysosomes, prions and oncogenes such as the H-Ras protein and oncogenic microRNAs [9–15]. Membrane nanotubes can also be utilized by natural killer cells for lysis of target cells [16]. These intercellular structures are also exploited for cell-to-cell transfer by pathogens [17–23] and evidence for TNT-like structures and TNTs *in vivo* has been provided in zebrafish embryos, in neural crest cells in chick embryos, in adult mouse cornea, as well as lung cancer biopsies [10, 24–28]. Recently, it was demonstrated that B-cell precursor acute lymphoblastic leukemia (BCP-ALL) cells and mesenchymal stem cells (MSCs) formed TNTs involving pro-survival cytokines and leukemic niche therapy resistance [29].

The exact molecular mechanisms responsible for TNT formation and regulation still remain elusive, however, molecules suggested to be important are; M-Sec (also called B94 or tumor necrosis factor-α inducing protein 2), the small GTPase RalA and the transmembrane protein leukocyte specific transcript 1 (LST1) [30, 31].

Here, we intended to study the existence, potential function and molecular mechanisms involved in TNT formation in AML cells and in addition investigate the effects of conventional AML chemotherapy on TNT formation.

## RESULTS

### TNT formation in primary AML cells

**Figure 1:**
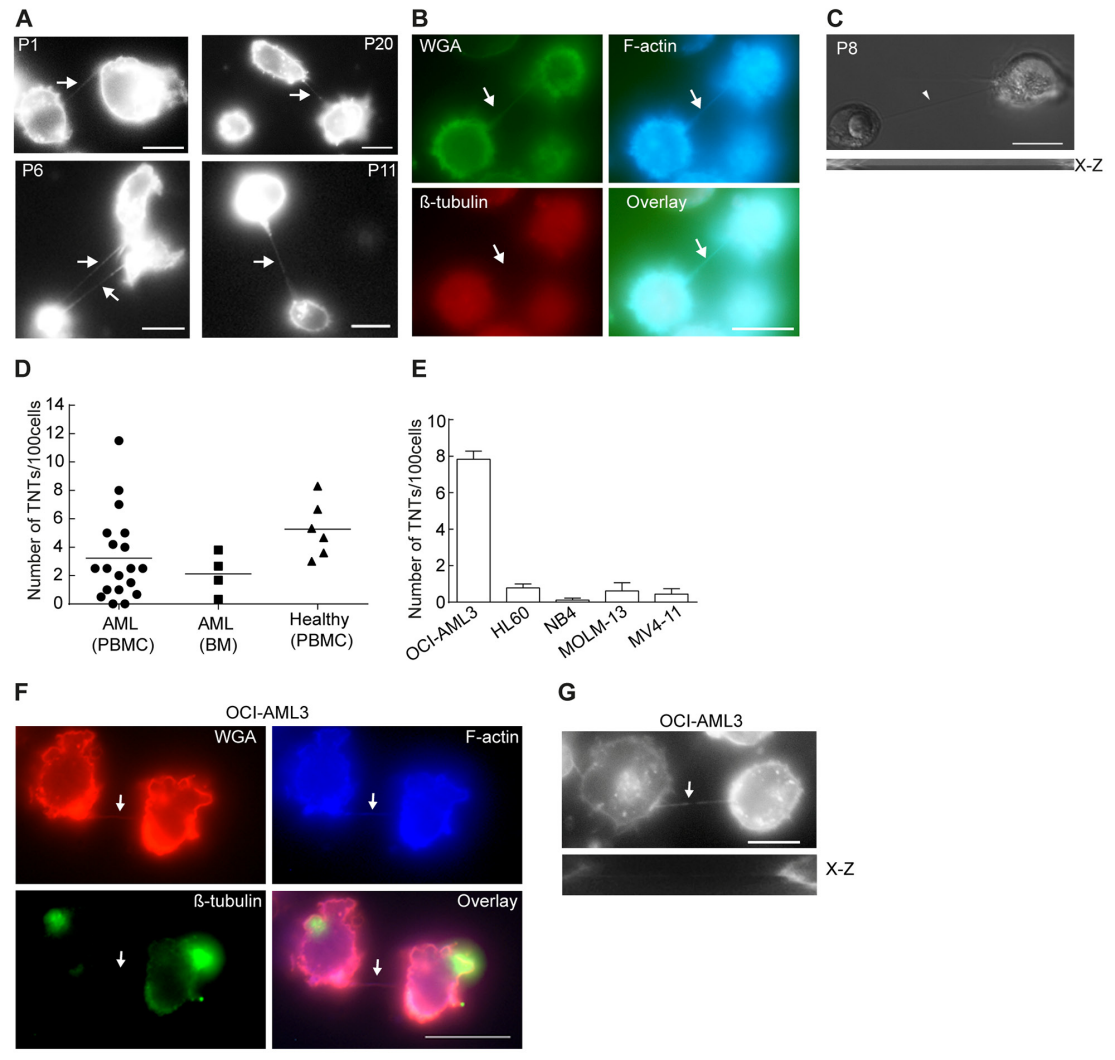
TNTs in primary AML cells and AML cell lines **A**. Representative images showing TNT connections (arrows) in live primary AML cells; peripheral blood-derived (P1, P6, P11) or bone marrow-derived cells (P20) stained with WGA-Alexa 594 and imaged by fluorescence and DIC microscopy. **B**. Fixed AML patient cells (P12) were stained with WGA-Alexa 488 (green), phalloidin (blue) and β-tubulin (red) and analyzed by fluorescence microscopy. Live AML patient cells were stained with WGA-Alexa 594 and TNTs were investigated by fluorescence and DIC microscopy. **C**. Representative image of AML cells (P8) with a TNT (arrow) not attached to the substrate (*x-z* scan, lower panel). **D**. Quantification of TNT connections in live WGA-Alexa 488 or 594 stained cells isolated from peripheral blood (PBMC) (*n=19*) and bone marrow (BM) (*n=4*) from AML patients or from peripheral blood (PBMC) of healthy blood donors (*n=6*). 100 cells were counted by fluorescence and DIC microscopy in duplicate or triplicate. **E**. Quantification of TNTs in live WGA-Alexa 488 or 594 stained AML cell lines. All cell lines were counted in duplicates (100 cells counted in each well) and repeated at least three times by fluorescence and DIC microscopy. Data are displayed as mean ± S.D. **F**. OCI-AML3 cells were stained with phalloidin Alexa 350 for F-actin labeling, antibodies against β-tubulin (mouse) followed by Alexa 488 goat-anti-mouse secondary antibody and WGA-Alexa 594. Arrows indicate TNT. **G**. Representative image of a TNT (arrow) connecting two OCI-AML3 cells not attached to the substrate (*x-z* scan, lower panel). Images were captured by AxioObserver Z1 microscope (Zeiss) using the 63x oil objective and analyzed by the ZEN 2012 software. All figures were created using Adobe Photoshop CS6 13.01 and Illustrator CS6 13.01. All Scale bars = 10 µm.

To study the existence of TNTs in primary AML cells, patient-derived leukemic cells were investigated by live fluorescence microscopy and TNTs were characterized according to the definition described in material and methods. Intercellular connection resembling TNTs were found in 17 of 19 patient-derived AML cells originating from peripheral blood and all four bone marrow-derived samples (Table 1). These intercellular connections spanned from 10-100 μm and one connection per cell was most common, however, some cells exhibited more than one (Figure 1A, P6). To verify that these connections indeed were TNTs, we confirmed the presence of F-actin, lack of tubulin and no attachment to the substrate as demonstrated by an *x-z* plane image (Figure 1B and 1C). Also, connections appearing after cell division, resembling TNTs, called cytoplasmic bridges were excluded by identification of their characteristic midbody by DIC. TNTs in peripheral blood-derived primary AML cells were found at a range between 0-11.5 TNTs/100 cells (Figure 1D, Table 1). TNT numbers in bone marrow-derived AML samples ranged from 0.33-3.8 TNTs/100 cells (Figure 1D, Table 1) and 3-8 TNTs/100 cells in peripheral blood mononuclear cells from six healthy individuals (Figure 1D). Intercellular structures more than 200 nm in diameter containing actin and tubulin were frequently observed, though not included in the TNT quantification.

**Table 1:** Characteristics of primary AML patient samples

	Patient No.	Sex	Age	FAB	Cytogenetics	Prev. disease	FLT3	NPM1	TNTs/100 cells
**Peripheral blood**	1	F	67	M5	t(9,11),+19	*De novo*	wt	wt	4
	2	M	71	M2	Normal	Relapse	TKD	ins	5
	3	F	78	M0	Complex	*De novo*	wt	wt	5
	4	M	54	M5	Normal	*De novo*	wt	ins	0
	5	M	58	M5	Normal	*De novo*	wt	wt	8
	6	M	33	M1	Normal	*De novo*	wt	wt	11.5
	7	M	68	M1	Normal	*De novo*	wt	wt	0
	8	M	64	M5	Normal	*De novo*	wt	MutA	4,2
	9	M	24	M2	Complex	CML	wt	wt	0.7
	10	M	46	M1	Normal	De novo	wt	MutA	2.5
	11	M	72	M5	Normal	*De novo*	wt	MutA	2.5
	12	F	68	M5	Normal	*De novo*	wt	MutA	7
	13	F	79	M1	Normal	*De novo*	ITD	ins	2.5
	14	M	42	M5	Normal	*De novo*	ITD	ins	1.5
	15	M	20	M2	Normal	*De novo*	ITD	wt	2
	16	F	57	M4	Inv16	*De novo*	wt	wt	1
	17	F	77	M1/2	Normal	*MDS*	ITD	ins	0.5
	18	F	78	M1	Normal	*De novo*	ITD	ins	1
	19	F	79	NA	Normal	*De novo*	ITD	ins	2.5
**Bone marrow**	7*	M	68	M1	Normal	*De novo*	wt	wt	2.7
	20	F	47	M4	Trisomi 8, t(11;17), (q23:p11)	*De novo*	wt	wt	3.8
	21	M	65	M1	Normal	MDS	wt	wt	0.3
	22	M	60	M1	Normal	*De novo*	wt	wt	1.7

FAB = French-American-British classification; FLT3 = fms-related tyrosin kinase 3; ITD = internal tandem duplication; TKD = tyrosine kinase domain; wt = wild type; NPM1 = nucleophosmin 1; ins = 4 nucleotide insertion in *NPM1*; MutA = 4 nucleotide (TCTG) insertion in *NPM1*; ins = 4 nucleotide insertion in *NPM1* [51]; MDS = myelodysplastic syndrome; * indicates identical patient.

### TNTs in AML cell lines

**Figure 2:**
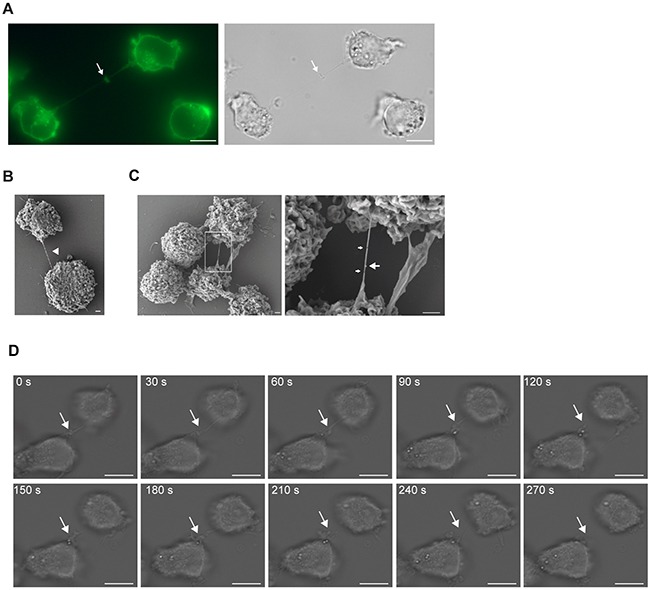
TNT formation in AML cell lines **A**. OCI-AML3 cells were stained with WGA-Alexa 488 and analyzed using fluorescence and DIC microscopy. Arrow indicates a cytoplasmic bridge characterized by a midbody. **B**. A representative image of a TNT connection (arrow head) between two OCI-AML3 cells captured by scanning electron microscopy. **C**. A representative image of a TNT connecting two OCI-AML3 cells and zoomed image (left, box). Right; small arrows indicate TNT fractures and large arrow indicates a knob. Images were captured by scanning electron microscopy Jeol JSM-7400F LEI 4.0 kV, x3000 (x3700 for D2) and WD 8.0 mm. **D**. Time-lapse microscopy of TNT formation in OCI-AML3 cells. Images were captured every 30^th^ sec by DIC microscopy. Arrow indicates the TNT. All images except scanning electron micrographs were captured by AxioObserver Z1 microscope using the 63x oil objective and analyzed by the ZEN 2012 software. All figures were created using Adobe Photoshop CS6 13.01 and Illustrator CS6 13.01. All Scale bars = 10 µm, except E-F = 1 µm.

Two different AML cell lines have previously been shown to express TNTs; THP-1 cells, not quantified per 100 cells, but demonstrated used for transfer of calcium flux and dyes [32] and KG1a cells found to have 13.97 ± 3.31 TNTs per 100 cells and shown to transport the stem cell marker CD133 [33]. To further investigate the function of TNTs in AML and search for potential key proteins involved in TNT formation, five distinct AML cell lines (Table 2) were examined for the presence of TNTs. However, except OCI-AML3 (7.8 TNTs/100 cells), the other cell lines expressed low numbers (0.1-0.8 TNTs/100 cells) of intercellular TNT-like structures (Figure 1E, Table 2). The intercellular structures found connecting OCI-AML3 cells were further verified as TNTs (Figure 1F and 1G) and cytoplasmic bridges were excluded by identification of their characteristic midbody (Figure 2A). Scanning electron microscopy (SEM) confirmed connections by thin (approximately 100 nm in diameter) structures between OCI-AML3 cells, not attached to the surface (Figure 2B, arrowhead). The fragility of the TNTs was illustrated by several fractures (Figure 2C, small arrows). A knob-like structure was observed in the TNT (Figure 2C, large arrow), similar to structures previously described involved in cell-to-cell transport [34].

**Table 2:** Cell line characteristics

Cell line	FAB	TP53	FLT3	NPM1	TNTs/ 100 cells
HL60	M2	del	wt	wt	0.8
MV4-11	M5	wt	ITD	wt	0.1
MOLM-13	M5	wt	ITD	wt	0.6
NB4	M3	mut	wt	wt	0.4
OCI-AML3	M4	wt	wt	MutA	7.8

FAB = French-American-British classification; FLT3 = fms-related tyrosin kinase 3; ITD = internal tandem duplication; wt = wild type; del = deletion; NPM1 = nucleophosmin 1; mut = mutated; MutA = 4 nucleotide (TCTG) insertion in NPM1 [51].

Time-lapse microscopy (intervals of 30 sec) of OCI-AML3 cells was used to examine how these TNTs formed and we found that they originated from filopodia-like structures which transformed into TNTs in less than three minutes (Figure 2D, Supplementary Video 1), a mechanism for TNT formation consistent with previous reported findings [8].

### Molecular mechanisms for TNT formation in AML cells

**Figure 3:**
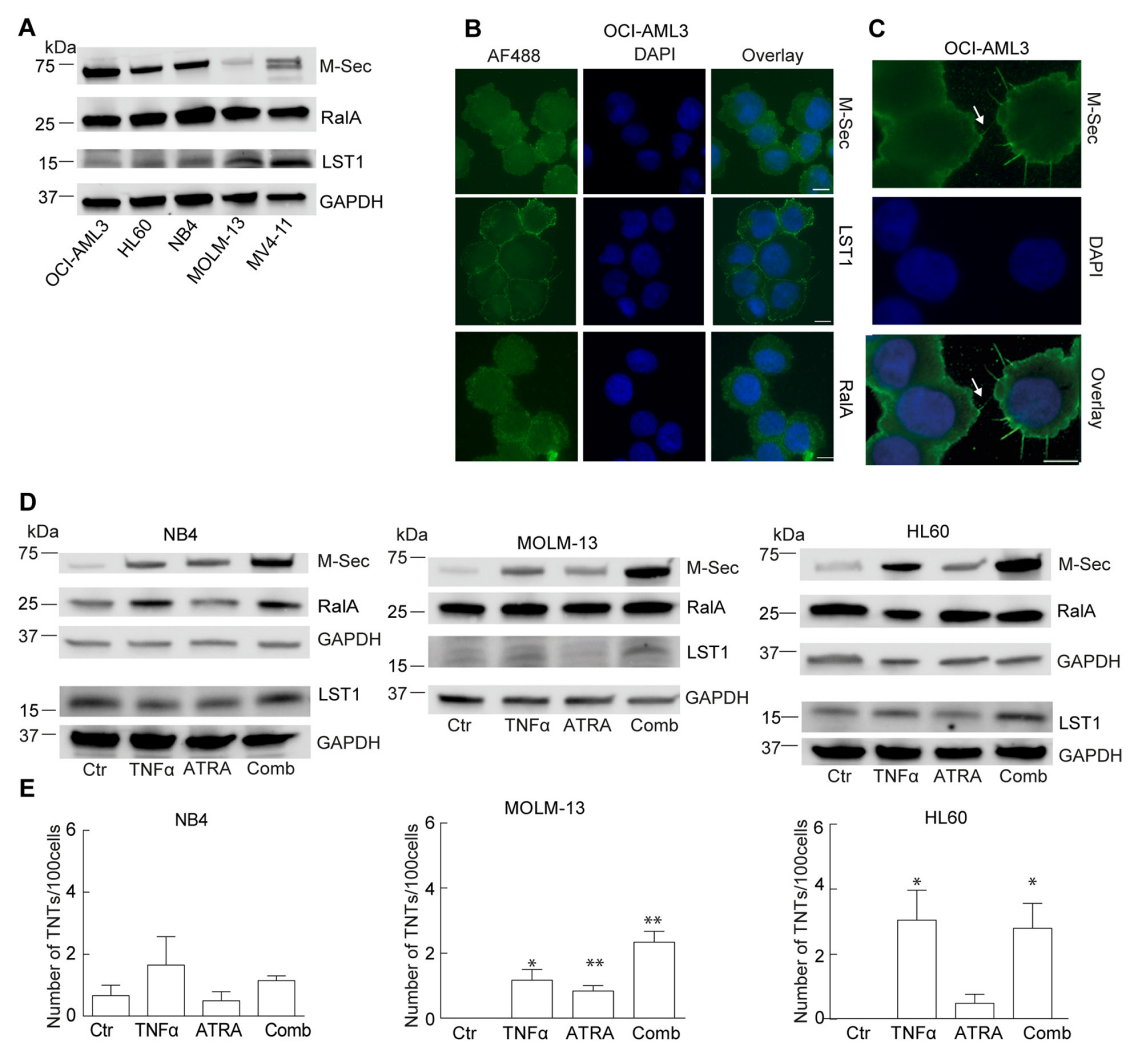
Investigation of the molecular machinery in TNT development in AML cell lines **A**. AML cells were lysed and subjected to immunoblotting using antibodies against M-Sec, RalA, LST1 and GAPDH (loading control). Representative blots from three independent experiments are shown. **B**. OCI-AML3 cells were fixed and immunostained with antibodies against M-Sec, LST1 and RalA and analyzed by fluorescence microscopy. **C**. Arrow indicates localization of M-Sec in TNT-like structures. **D**. NB4, MOLM-13 and HL60 cells were treated for 24 h with 0.15% EtOH (Ctr), ATRA (10 µM) or TNFα (10 nM) alone or in combination and immunoblotted using antibodies against M-Sec, RalA, LST1 and GAPDH as loading control. Representative blots from three independent experiments are shown. **E**. Quantification of TNTs in NB4, MOLM-13 and HL60 cells (*n = 3*) (*P < 0.05, **P < 0.01). All scale bars = 10 µm.

The molecular mechanisms behind TNT formation and TNT-mediated transport are mostly unknown. In myeloid cells, three proteins; M-Sec, RalA and LST1, have previously been reported to be central in TNT formation [30, 31] and we therefore investigated their role in TNT formation in AML cells. All the five AML cell lines studied endogenously expressed M-Sec, however, the TNT abundant OCI-AML3 cell line expressed highest M-Sec protein levels (Figure 3A). The RalA protein was highly and similarly expressed in all the AML cell lines whereas LST1 showed limited protein expression, with lowest amounts found for the OCI-AML3 cells (Figure 3A). We further examined the subcellular localization of M-Sec, RalA and LST1 in OCI-AML3 cells by immunofluorescence. All three proteins mainly localized to the cytoplasm and LST1 was most prominently localized to the plasma membrane (Figure 3B). M-Sec was also found localized to the TNT structure itself and was often associated with filopodia-like protrusions (Figure 3C, arrow). In the other cell lines, M-Sec, RalA and LST1 mostly localized to the cytoplasm, and only MV4-11 cells demonstrated evident localization of LST1 to the plasma membrane (data not shown).

Since four of five AML cell lines expressed limited TNT numbers (Figure 1E) and variable M-Sec protein levels (Figure 3A), we sought to examine if induction of endogeneous M-Sec expression could result in increased TNT numbers. The A-vitamin derivate all-trans retinoic acid (ATRA), used in the treatment of acute promyelocytic leukemia (APL), has previously been shown to upregulate M-Sec at the mRNA level alone and in combination with tumor necrosis factor α (TNFα) [35]. We therefore treated the APL cell line NB4 and the two non-APL cell lines; HL60 and MOLM-13, with TNFα (10 nM) or ATRA (10 μM) for 24 h separately or in combination. TNFα treatment increased the M-Sec level in all three cell lines; ATRA treatment also caused an increase in M-Sec, most prominent in NB4 cells, however, most evident was the increase in M-Sec after combining these two compounds (Figure 3D, Supplementary Figure 1), as also demonstrated earlier [36]. Treatments caused only minor changes in RalA protein level in the three cell lines, whereas a minor increase in LST1 protein levels was observed in TNFα-treated MOLM-13 cells and in TNFα and ATRA treated NB4 cells (Figure 3D, Supplementary Figure 1). TNT numbers were modestly induced by TNFα treatment, however, the TNFα and ATRA combination, resulting in the highest increase in M-Sec levels, was not associated with an additional increase in TNTs (Figure 3E).

**Figure 4:**
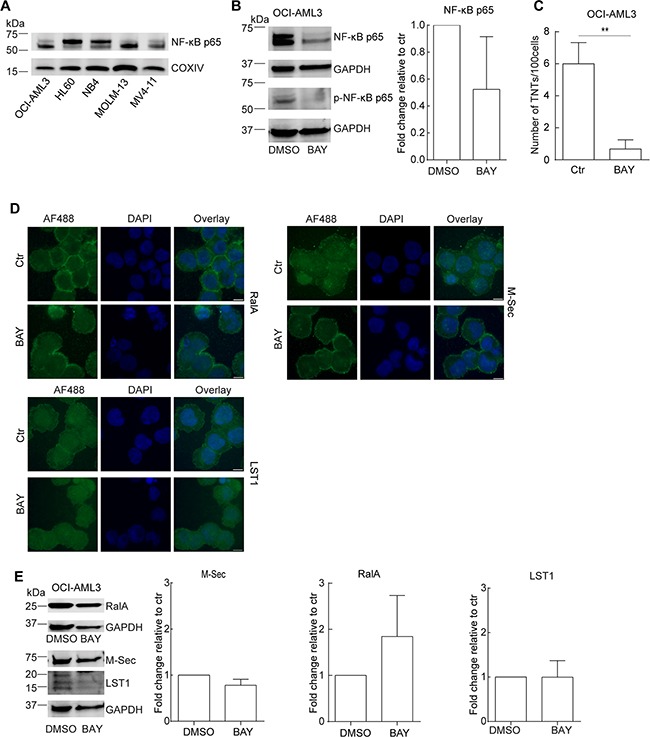
The NF-κB pathway is involved in TNT formation in AML cells **A**. AML cell lines were lysed and immunoblotted using antibodies against NF-кB-p65 and COXIV as loading control. Representative blots from three independent experiments are shown. **B**. OCI-AML3 cells were treated with BAY 11-7082 (2.5 µM, 24 h) and analyzed by immunoblotting with antibodies against NF-кB p65 and NF-кB p-p65. Representative blots from three independent experiments are shown. **C**. TNT quantification of OCI-AML3 cells treated with BAY 11-7082 (2.5 µM, 24 h) and control (*n = 3*) (**P < 0.01). **D**. OCI-AML3 cells were treated with BAY 11-7082 (2.5 µM, 24 h) and analyzed by immunofluorescence with antibodies against RalA, M-Sec and LST1. **E**. OCI-AML3 cells were treated with BAY 11-7082 (2.5 µM, 24 h) and immunoblotted with antibodies against RalA, M-Sec and LST1 with additional GAPDH as a loading control. Representative blots from three independent experiments are shown in addition to the quantification. Scale bars = 10 μm. Data are displayed as mean ± S.D.

Expression of the *M-Sec* gene is directly induced by TNFα [37] and in AML cells, the TNFα–NF-κB pathway is frequently constitutively active correlating with chemoresistance [38, 39]. In order to further investigate the role of M-Sec in TNT formation in AML cells, we compared the basal expression level of the NF-κB p65 protein in different AML cell lines by immunoblotting. This revealed that the AML cell lines expressed diverse levels of p65 protein (Figure 4A). Since the TNT and M-Sec abundant OCI-AML3 cells expressed high levels of the p65 protein we decided to investigate the effect of the small molecular NF-кB inhibitor BAY 11-7082 [40]. OCI-AML3 cells were treated for 24 h with a pre-apoptotic concentration (2.5 μM, 6% cell death) which resulted in a reduction of total protein levels of total p65 and phospho-p65 (Figure 4B). By immunofluorescence, the p65 and p-p65 proteins mainly localized to the cytoplasm, but also in a dotted pattern in the nucleus and BAY 11-7082 treatment did not induce a change in subcellular localizations, however, a modest reduction in fluorescence intensity was observed (data not shown). BAY 11-7082 treatment resulted in a significant reduction in the number of TNTs (Figure 4C), however, no changes in the subcellular localization of M-Sec, RalA or LST1 were seen, but a minor decrease in M-Sec and an increase in RalA protein levels were found (Figure 4D,E). This indicated an involvement of the NF-κB pathway in TNT formation in OCI-AML3 cells, where an association with the M-Sec and RalA proteins could not be excluded.

**Figure 5:**
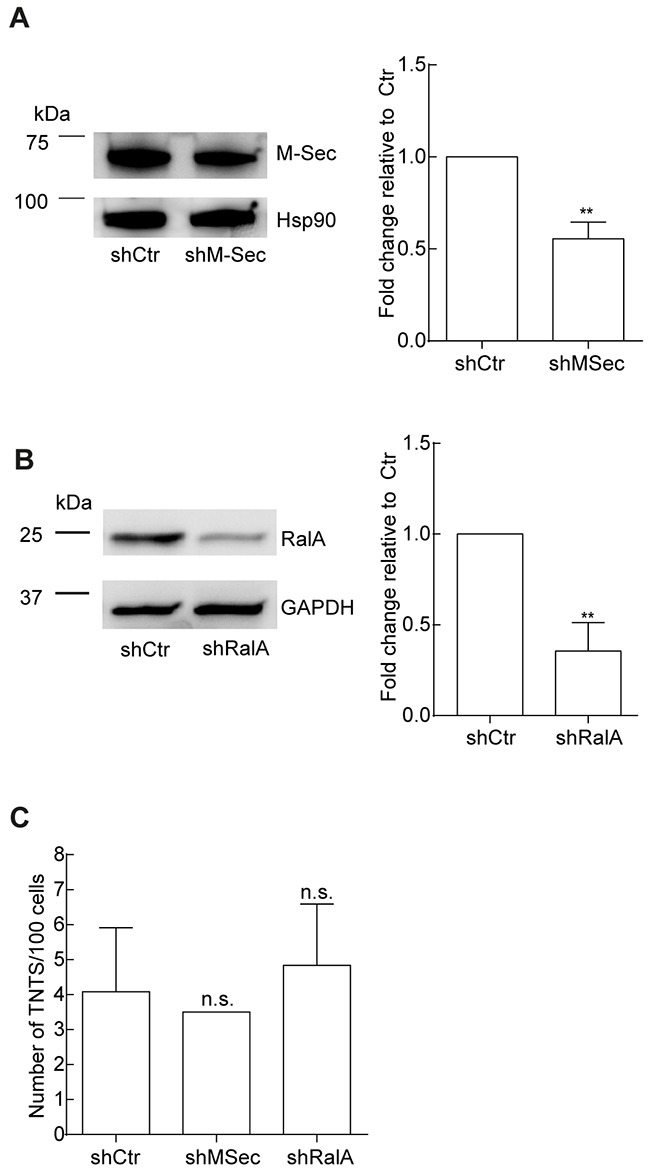
The role of M-Sec and RalA in TNT formation in OCI-AML3 cells **A-B**. OCI-AML3 cells transduced with shCtr, sh-M-Sec and shRalA. Representative immunoblots of three independent experiments are shown for verification of shRNA knock-down of M-Sec (A) and RalA (B). Immunoblots were quantified with Image Quant TL with significant changes indicated (**P < 0.01). **C**. TNT quantifications were performed in parallel with the three independent immunoblot experiments in A-B. Data are displayed as mean ± S.D. n.s. = not significant

Based on this we further investigated the potential role of M-Sec and RalA in TNT formation and performed a knock-down by shRNA against M-Sec and RalA, verified and quantified by immunoblotting relative to shCtr (Figure 5A, 5B). The efficiency of the knock-down was found to be approximately 40% for M-Sec and approximately 60% for RalA, relative to the shCtr. When these cells were compared with respect to TNT formation, no significant differences were found (Figure 5C).

### The effect of AML standard induction chemotherapy on TNTs

**Figure 6:**
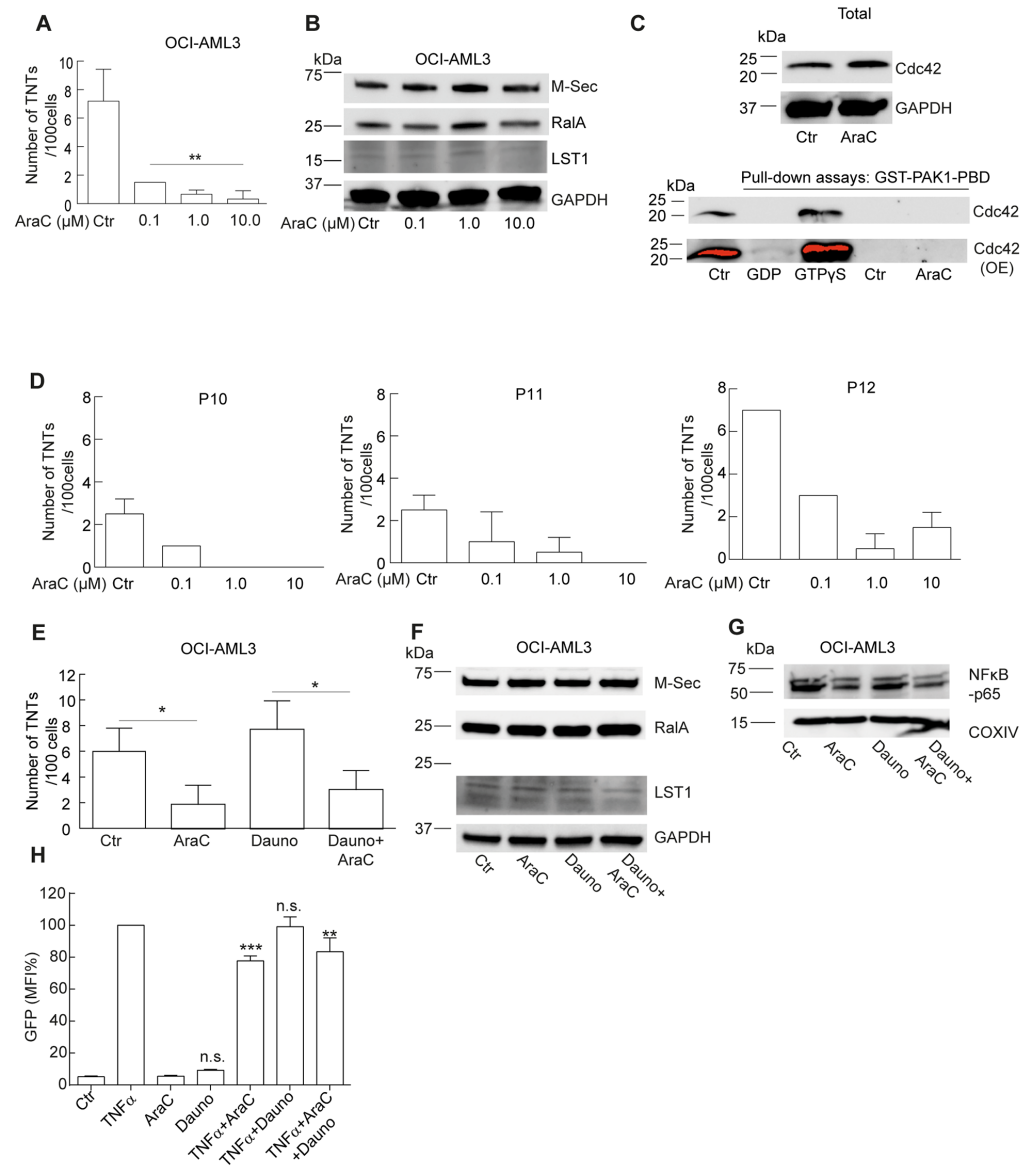
Cytarabine downregulates TNT formation in AML cells **A**. OCI-AML3 cells were treated with 0.1, 1.0 and 10 µM cytarabine (AraC) for 24 h and TNTs were quantified; (*n = 3*, **P < 0.01). **B**. OCI-AML3 cells were treated with 0.1, 1.0 and 10 µM cytarabine (AraC) for 24 h and immunoblotted with antibodies against M-Sec, LST1 or RalA; representative blots from three independent experiments are shown. **C**. OCI-AML3 cells were untreated (Ctr) or treated for 1 h with cytarabine (AraC) and immunoblotted using antibodies against Cdc42 and GAPDH as a loading control. Pull-down assay using GST-PAK1-PBD on cell lysates from OCI-AML3 cells untreated (Ctr) or treated for 1 h with cytarabine (AraC) where GDP and GTPγS represent the negative and positive control, respectively. Immunoblotting for detection of active (GTP-bound) Cdc42 was performed with an antibody against Cdc42. OE = overexpressed. **D**. Density gradient separated PBMCs from three AML patients (P10, P11, P12) were treated *in vitro* with 0.1, 1.0 and 10 µM AraC for 24 h. The experiment was performed in duplicates. **E**. OCI-AML3 cells treated with 1.0 µM AraC, 100 nM daunorubicin alone or in combination for 24 h and quantified for TNTs (*n = 3*) (*P < 0.05). **F**. OCI-AML3 cells treated with 1.0 µM AraC, 100 nM daunorubicin alone or in combination for 24 h, lysed and immunoblotted using antibodies against M-Sec, RalA, LST1 and **G.** NF-кB-p65. Representative blots from three independent experiments are shown. **H**. NF-κB-GFP-Jurkat cells treated without or with TNFα (5 ng/ml); with or without cytarabine (1 μM) or daunorubicin (100 nM) for 24 h before analyzed live by flow cytometry. (n=3) Data are displayed as mean ± S.D. n.s. = not significant.

The standard induction treatment of AML patients includes cytarabine (AraC) in combination with an anthracycline (idarubicin or daunorubicin) [41]. Interestingly, cytarabine has been reported to have a negative effect on the NF-κB pathway and daunorubicin a positive [42, 43], it was therefore of interest to study the effect on TNTs of these drugs separately and in combination. OCI-AML3 cells were treated for 24 h with cytarabine in three different concentrations (0.1 µM, 1.0 µM and 10 µM), reflecting *in vivo* treatment with low (20 mg/m^2^. BID), intermediate (200 mg/m^2^ every 18 h) and high dose (3 g/m^2^, BID) cytarabine therapy [44]. Treatment for 24 h with these three concentrations resulted in a reduction in TNTs, where induction of cell death was negligible or low (0%, 2% and 10%, Figure 6A). Cytarabine-treated OCI-AML3 cells did not indicate any change in M-Sec, RalA or LST1 protein levels (Figure 6B). Since we found that TNTs in OCI-AML3 cells could be formed through filopodia interplay and the GTPase Cdc42 (22 kDa) has been associated with filopodia formation, actin remodeling and TNT formation [30, 45], we investigated the Cdc42 activity in OCI-AML3 cells, compared to OCI-AML3 cells treated with cytarabine for 1 h. This was performed by the use of a GST1-PAK1(p21-activated kinase)-PBD pull-down assay. Activated Cdc42 (GTP-bound, not GDP-bound) interacts with a protein binding domain (PBD) at the N-terminal of PAK resulting in PAK activation and actin reorganization [46]. OCI-AML3 cells were found to express the Cdc42 protein and no reduction was apparent after cytarabine treatment (Figure 6C). Active and inactive Cdc42 was verified in the pull-down assay by the use of GTPγS and GDP, however, no active Cdc42 was found in the OCI-AML3 cells, even after overexposing (OE) the membrane (Figure 6C). Another GTPase also interacting with PAK is the Rac1 (also 22 kDa) protein [46]. Based on this we attempted to incubate the same membrane with an anti-Rac1 antibody and surprisingly, bands appeared indicating active Rac1 in the OCI-AML3 cells, however, with no apparent change after cytarabine treatment (Supplementary Figure 2A). TNTs were then quantified in three AML patient samples (P10, P11, P12), with similar molecular characteristics (Table 1), treated with cytarabine (0.1 µM, 1.0 µM and 10 µM, 24 h) also resulting in a reduction in TNTs, confirming the downregulating effect of cytarabine (Figure 6D). A minor reduction in TNTs was also found in PBMCs obtained from an AML patient (P8, Table 1) at diagnosis versus day 17 after chemotherapy with cytarabine (days 1-7) and daunorubicin days (1-3) (Supplementary Figure 2B). Treatment of OCI-AML3 cells for 24 h with pre-apoptotic doses [47] of daunorubicin (100 nM) did not significantly affect TNT numbers; however, daunorubicin combined with cytarabine (1 μM) resulted in reduced TNTs (Figure 6E). The protein levels of M-Sec, RalA or LST1 did not significantly change within the 24 h of chemotherapy treatment as analyzed by immunoblotting (Figure 6F, Supplementary Figure 2C). However, we did find that cytarabine reduced p65 expression alone and in combination with daunorubicin (Figure 6G). To further elucidate the effect of cytarabine and daunorubicin on the NF-κB pathway, we utilized a NF-κB-GFP-Jurkat reporter cell line, where NF-κB-dependent GFP expression was measured by flow cytometry. TNFα treatment (5 ng/ml), used as a positive control, induced GFP expression, whereas no significant differences were found following cytarabine or daunorubicin treatment alone (Figure 6H). In combination with TNFα treatment; cytarabine alone and in combination with daunorubicin significantly reduced GFP expression, whereas no change was found after treatment with daunorubicin alone (Figure 6H).

### Daunorubicin is transported between cells through TNTs

**Figure 7:**
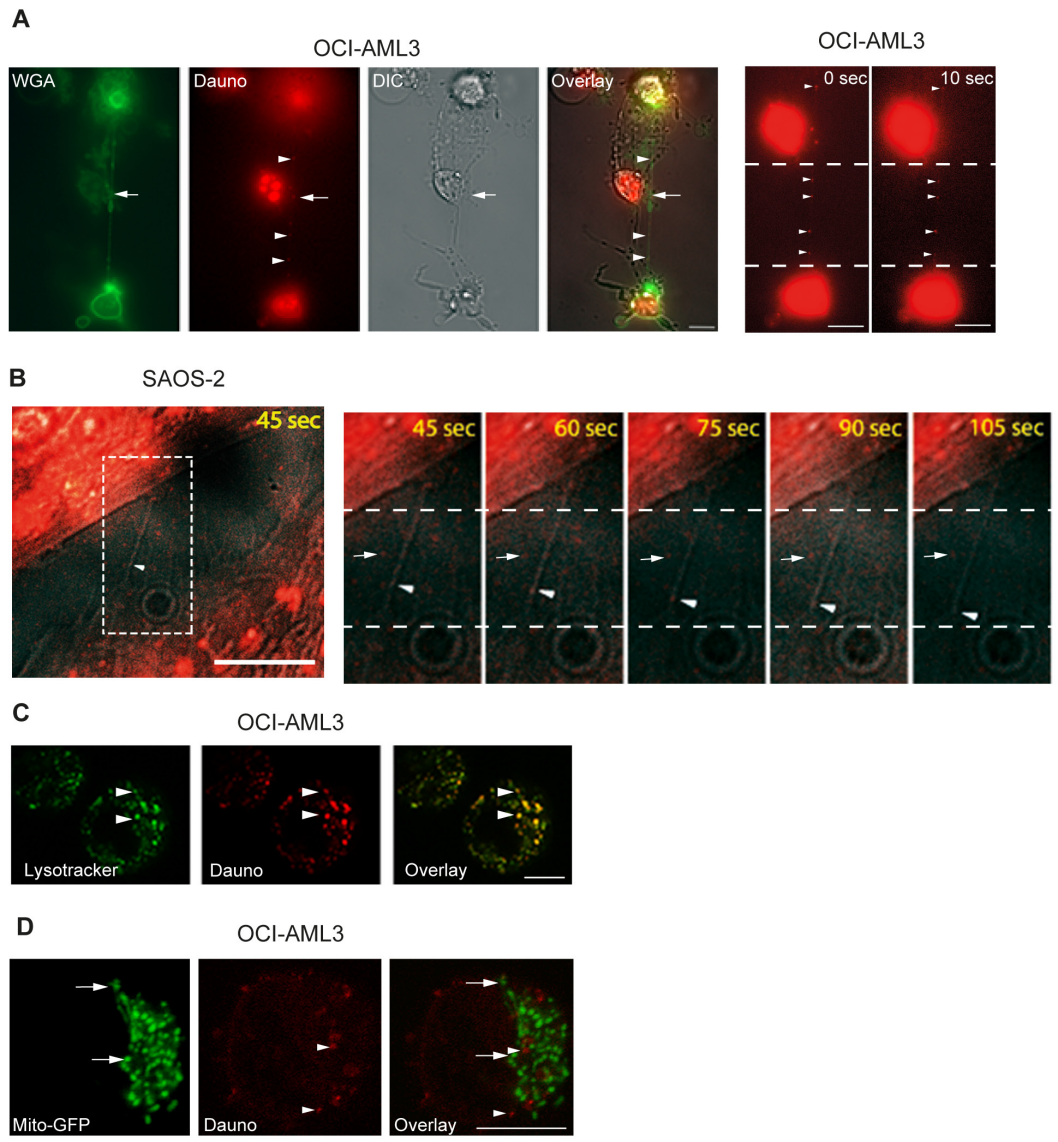
Cell-to-cell transfer of daunorubicin by TNTs **A**. OCI-AML3 cells were treated with 100 nM daunorubicin (fluorescently red, Dauno) for 24 h, stained with WGA-Alexa 488 (green) and investigated by live fluorescence and DIC microscopy. Arrow indicates TNT structure and arrowheads indicate localization of daunorubicin. Images are representatives of three independent experiments. Zoomed image and a second image captured 10 sec later, arrowheads indicate daunorubicin. **B**. SAOS-2 cells were treated with 100 nM daunorubicin for 2 h, stained with WGA-Alexa 488 and analyzed by live fluorescence microscopy. Images were captured every 15^th^ sec using DIC and the red channel only in order to reduce total time of light exposure. Zoomed images (from white box) illustrate daunorubicin transport (arrowheads) compared to daunorubicin with no movement (arrow). **C**. OCI-AML3 cells treated with 100 nM daunorubicin (red) overnight and stained with 1 µM Lysotracker-Green (green) for 2 h before investigation by confocal fluorescent microscopy. Arrow heads indicate colocalization between lysotracker and daunorubicin. **D**. Mito-GFP (green, arrows) stably expressing OCI-AML3 cells were treated with 100 nM daunorubicin (red, arrowheads) for 24 h and investigated by confocal microscopy. Confocal microscopy was performed by Zeiss LSM 500 META. All scale bars = 10 µm.

Interestingly, daunorubicin (being fluorescent) localized to the TNTs connecting OCI-AML3 cells and in 10 sec we observed that daunorubicin slightly moved in the TNT (Figure 7A, red dot). Since OCI-AML3 cells are suspension cells, they are only semi-adherent in the fibronectin-coated well and therefore cellular movement during the time-lapse experiment is inevitable. Because of this and the extreme fragility of the TNTs during imaging capturing we did not succeed in generating a video displaying the TNT transport of daunorubicin. To investigate the potential transport we therefore utilized the adherent osteosarcoma cell line SAOS-2 previously shown to have TNT forming capabilities [48]. Images were taken every 15^th^ sec and daunorubicin was found to be transported in the TNTs (Figure 7B and Supplementary Video 2). As an illustration of the fragility of these structures, notice the longer TNT, indicated by an arrow, being stretched and then break in comparison to the two shorter TNTs, also indicated by arrows (Supplementary Video 3).

**Figure 8:**
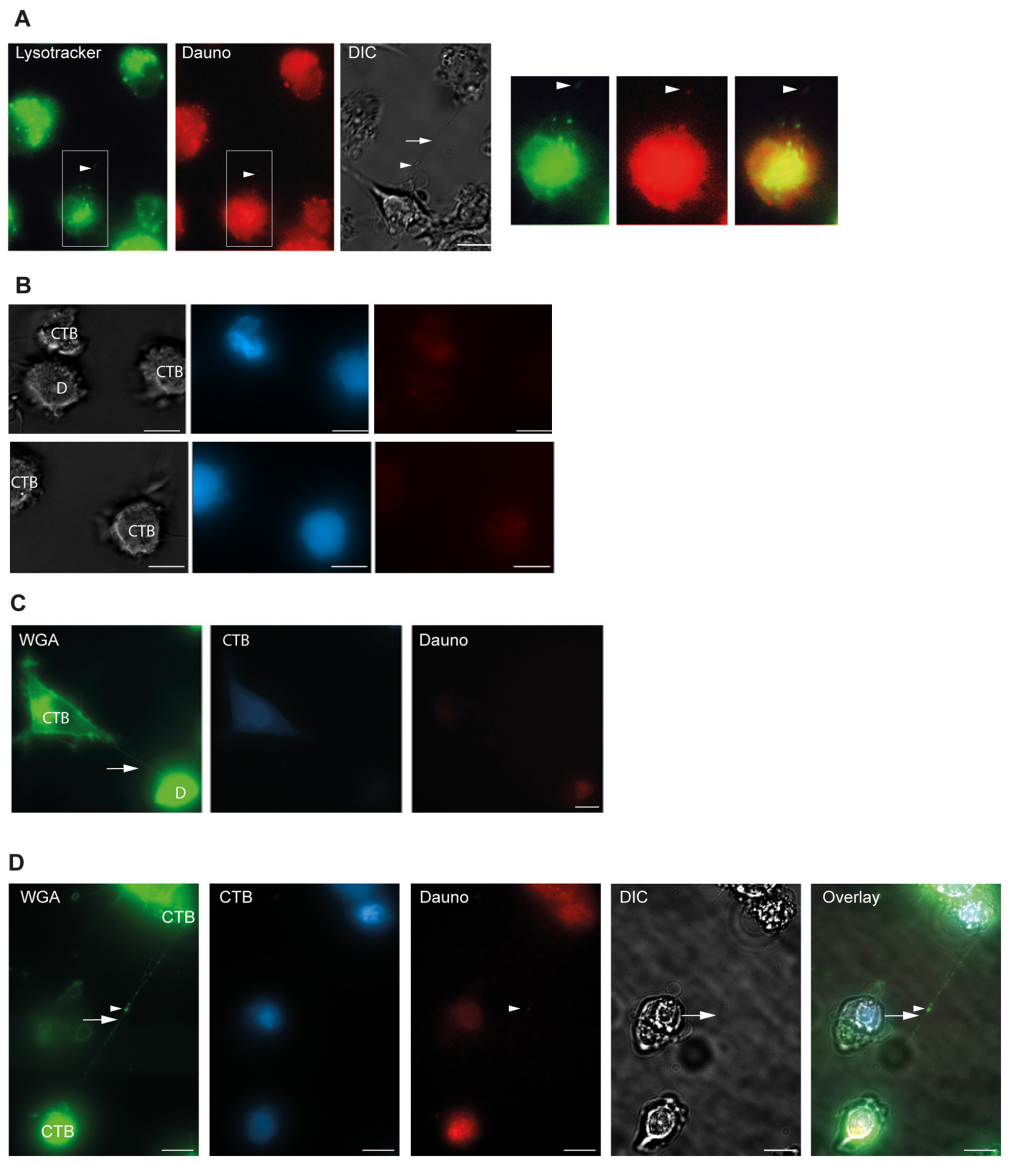
Daunorubicin is localized in lysosomes **A**. OCI-AML3 cells were stained with 1 µM lysotracker Green and treated with 100 nM daunorubicin (red) for 24 h and investigated by fluorescent microscopy and DIC. Arrowheads show colocalization of lysotracker Green, daunorubicin and TNT (arrow, DIC). **B**. Co-culture of cell tracker blue (CTB) stained cells and daunorubicin (D) stained cells. **C**. TNT connection (arrow) between untreated OCI-AML3 cells stained with cell tracker blue (CTB) and OCI-AML3 cells treated with 100 nM daunorubicin. **D**. Untreated CTB-positive cells form TNT containing daunorubicin. Daunorubicin positivity occurs by uptake from cell culture medium. Co-culture experiments (*n=2*). Scale bars = 10 µm.

Since daunorubicin is associated with cellular membranes and lysosomes [49, 50], and lysosomes themselves can be transported through TNTs [8], we labeled OCI-AML3 cells with the lysosomal specific dye Lysotracker-Green before treatment with daunorubicin for 24 h. Indeed, we found that daunorubicin colocalized with lysosomes in the OCI-AML3 cells (Figure 7C) whereas no colocalization was found between daunorubicin and mitochondria in OCI-AML3-mito-GFP cells (Figure 7D). Strikingly, daunorubicin and Lysotracker-Green also colocalized within the TNTs connecting OCI-AML3 cells (Figure 8A), demonstrating that daunorubicin can be transported in TNTs through interaction with lysosomes. Further, in co-culture experiments between Celltracker-Blue labeled cells (CTB, untreated) and daunorubicin treated cells (Figure 8B) we found that untreated and treated cells formed TNTs (Figure 8C). Interestingly, untreated cells (Celltracker-Blue) that turned daunorubicin positive, formed TNTs with surrounding untreated cells, where daunorubicin localized to the TNTs (Figure 8D). This indicated that untreated cells receiving daunorubicin were capable of forwarding this drug to neighboring cells.

## DISCUSSION

This study demonstrates for the first time the presence of TNTs in primary AML samples derived from peripheral blood and bone marrow. The TNT numbers in the patient-derived samples showed a greater variation compared to cells from healthy blood donors. However, the TNT numbers were in general low as only 3 of total 22 patient samples had TNT numbers above 5 TNTs/100 cells and in 12 of 19 blood-derived patient samples, TNT numbers were lower than 3 TNTs/100 cells. Also, 17 of these patient samples had a normal karyotype and interestingly, all patient samples containing *FLT3-ITD* mutations expressed 2.5 or less TNTs/100 cells. In comparison, the primary samples with *NPM1* mutation [51] only, showed a greater variation. As for the AML cell lines, all except the *NPM1***-**mutated OCI-AML3 cells, demonstrated very low TNT numbers. In order to further correlate TNT numbers and AML associated molecular characteristics, additional AML patient samples need to be included, however, thus far AML cells appear to be associated with relatively low TNT numbers.

Although, the molecular mechanisms responsible for TNT formation are not elucidated, a limited number of proteins that directly or indirectly associate with actin polymerization have been suggested to be central [52, 53]. These proteins include Myo10, the calcium binding protein S100A4 and its receptor RAGE in neurons and astrocytes, and the small GTP binding protein Cdc42, together with M-Sec, RalA, LST1, filamin and the exocyst complex, particularly in macrophages, leukocytes and in an overexpression system in HeLa cells [52, 53]. Since M-Sec, LST1 and RalA all are expressed in the myeloid lineage; they were chosen to assess their role in TNT formation in AML cells. We found that the most TNT abundant cell line OCI-AML3 also expressed highest amounts of M-Sec protein. Since M-Sec is induced by TNFα through a NF-κB site identified in the M-Sec promoter [37, 40], and ATRA induces mRNA expression of M-sec through an TNFα independent mechanism in NB4 and HL60 cells [35]; we used these two compounds to increase endogenous M-Sec expression in the low number TNT expressing cells NB4, MOLM-13 and HL60. TNFα treatment alone did increase TNT numbers slightly; however, even if TNFα and ATRA increased endogenous expression levels of M-Sec protein more than additive, no further increase in TNT numbers were observed. These results suggested that in AML cells TNFα-induced NF-κB activation could play a role in TNT regulation. This was further confirmed by the use of the NF-κB inhibitor BAY-117082 which downregulated both NF-κB p65 and phospho-p65 protein expression together with a pronounced reduction in TNT numbers. We also found a modest decrease in the protein expression of M-Sec and an increase in RalA. However, knock-down of M-Sec and RalA did not appear to have any significant effects on TNT formation in OCI-AML3 cells and this suggested a role for the NF-κB pathway in TNT regulation in AML cells.

This was further supported by treatment of AML cells with the standard induction therapy cytarabine and daunorubicin [54, 55]. Interestingly, daunorubicin has been associated with increased NF-κB activation [42], whereas cytarabine has been demonstrated to inhibit this pathway [43]. In OCI-AML3 cells and in three AML patient samples (P10, P11, P12, Table 1) we observed that cytarabine caused a significant reduction of TNTs and NF-κB p65 protein expression level, with again no difference in the protein levels of M-Sec, RalA or LST1. Daunorubicin treatment alone did not decrease TNTs, however, in combination with cytarabine TNTs were reduced to similar levels as with cytarabine alone. A reduction in TNT numbers was also found in cells obtained from an AML patient (P8, Table 1) after treatment with cytarabine and daunorubicin. The effects on NF-κB by daunorubicin and cytarabine were verified by treatment of the NF-κB-GFP-Jurkat reported cell line where basal level NF-κB induction was studied compared to effects following NF-κB activation by the use of TNFα. We reproduced the NF-κB downregulating effect of cytarabine by the use of much lower concentrations than the ones used by Screenivasan and co-workers also demonstrating this effect in Jurkat cells [43]. However, we did not find that daunorubicin significantly activated NF-κB at the basal level or further after induction with TNFα and this could be due to differences in concentrations used as well as cell types; we used 100 nM daunorubicin in OCI-AML3 cells and Das & White used from 2-5 μM in the human lung adenocarcinoma cell line A549 [42]. This demonstrated that cytarabine is a NF-κB and TNT-downregulating drug in AML cells.

To further search for molecular mechanisms associated with these findings, we hypothesized that the RhoGTPase Cdc42, reported activated by NF-κB induction and having a central role in filopodia induction and actin reorganization [56], could be influenced in OCI-AML3 cells following cytarabine treatment. However, we were not able to demonstrate the presence of active (GTP-bound) Cdc42 in the OCI-AML3 cells, indicating that other RhoGTPases closely linked to NF-κB activation are involved in TNT formation in these cells [56, 57]. Therefore additional studies are needed to elucidate the exact molecular mechanisms involved in the TNT formation in AML cells.

Since daunorubicin itself is fluorescent it could readily be observed colocalized with lysosomes in OCI-AML3 cells. More strikingly, this drug colocalized with lysosomes in the TNT structure connecting OCI-AML3 cells, demonstrating that TNTs could function as intercellular transport devices of this drug. By the use of the adherent osteosarcoma cell line SAOS-2 and time-lapse microscopy, we demonstrated that daunorubicin was transported through TNTs. This could represent an approach for AML cells to communicate stress response to surrounding cells, possibly inducing collateral damage to the microenvironment.

Recently, Polak and co-workers demonstrated that B-cell precursor acute lymphoblastic leukemia (BCP-ALL) cells signals through TNTs to MSCs, reflecting the leukemic bone marrow niche, resulting in increased cytokine secretion which acted pro-survival and was associated with therapy resistance [29]. This study suggested that drugs targeting TNT communication could eliminate leukemic cells and prevent drug resistance. Our results indicate that TNT communication between AML cells could be rather limited in cells with *FLT3-ITD* mutation suggesting less survival dependency towards the stromal microenvironment as compared to AML cells with *NPM1* mutation only. Subsequently, we find that cytarabine and daunorubicin treatment downregulates TNT numbers, this could represent a contributing factor for the sensitivity towards induction therapy found for *NPM1*-mutated AML. This hypothesis needs to be tested using co-cultures with AML cells and MSCs similar to the above described study. In conclusion, TNT intercellular communication represents a potential mechanism for chemoresistance that should be examined in future therapy development.

## MATERIALS AND Methods

### Cell lines

MOLM-13, HL-60, NB4, MV4-11, SAOS-2 (ATCC) and OCI-AML3 (DSMZ) cells were cultured according to the provider's instructions. The Mito-GFP-OCI-AML3 cells were generated by transducing OCI-AML3 cells with ready-to-use lentiviral particles expressing a mitochondrial localization signal fused to GFP; rLV-EF1-AcGFP-Mito-9 (Takara, rV2.1A1.1941 C4) according to the provider's instructions. The shM-Sec, shRalA and shCtr OCI-AML3 cells were generated by transducing OCI-AML3 cells with ready-to-use lentiviral particles (Santa Cruz Biotechnology, sc-45826-V, sc-41842-V, sc-108080) according to provider's instructions. Initially, copGFP Control lentiviral particles (sc-108084) were used to investigate the efficiency of OCI-AML3 transduction using these ready-to-use lentiviral particles.

Post-transduction, cells were selected using 5 μg/ml puromycin for two weeks, using OCI-AML3 cells only as a control for puromycin induced cell death, before verification of shRNA effect by immunoblotting. The OCI-AML3 cells were transduced with shM-Sec and shRalA lentiviral particles separated in time and each transduction was accompanied by a transduction with shCtr. For the (*n=3*) TNT quantification the shCtr cells have been pooled and represents (*n=6*). The NF-κB-GFP-Jurkat reporter cell line was obtained from System Biosciences (SBI). All media were supplemented with 10% heat inactivated FBS, 1% 2 mM L-glutamine and 1% 5 mM (1.0 U/ml) penicillin and streptomycin (Sigma-Aldrich).

### Primary AML samples

The study was conducted in accordance with the Declaration of Helsinki and approved by the local Ethics Committee (Regional Ethics Committee West, University of Bergen, Norway). Blood and bone marrow samples from consecutively diagnosed AML patients with high peripheral blood blast counts (>7*10^9^/L) were collected after informed consent and were processed by density gradient separation (Lymphoprep, Axis-Shield, Oslo, Norway) with >95% leukemic blasts for biobanking as previously described [58].

The PBMCs were obtained from blood donors at the Blood Bank at Haukeland University Hospital following a written consent and isolated by density gradient separation.

### Antibodies and reagents

The following primary antibodies were used for immunofluorescence and/or immunoblotting: anti-β-tubulin (clone TUB 2.1, Sigma-Aldrich), anti-TNFα-IP2 (sc-28318, Santa-Cruz), anti-NF-кB-p65 (C22B4, Cell signaling), anti-NF-кB-P-p65 (S536, Cell signaling), anti-Hsp90 (SPA830, Stressgen, Enzo Life Sciences), anti-Rac1 (1862341, Thermo Fisher Scientific) from Abcam; anti-LST1 (LST1/02, ab81439), anti-RalA (EPR6468, ab126627), anti-COX IV (ab16056), anti-GAPDH (ab9485). Secondary antibodies used for immunofluorescence or immunoblotting; Alexa Fluor^©^ 488- or 594-conjugated goat-anti-mouse (Invitrogen), horseradish peroxidase (HRP)-conjugated goat anti-rabbit/mouse (Jackson Immunoresearch). The following were used for actin and membrane staining; AlexaFluor^©^ 350-conjugated phalloidin and wheat germ agglutinin (WGA)–Alexa Fluor^©^ 594 or 488 (Invitrogen), for co-culture experiments; Celltracker-Blue CMAC (7-amino-4-chloromethylcoumarin), Lysotracker-Green DND-26 (all from Invitrogen). Cytarabine (Pfizer) and daunorubicin (Galen Ltd.) were obtained from Western Norway Pharmaceutical Trust, tumor necrosis factor α (TNFα) and all-trans retinoic acid (ATRA) (Sigma-Aldrich) and BAY 11-7082 (Calbiochem).

### TNT identification and quantification

A TNT in this study is defined as; a thin straight structure, ≤ 200 nm in diameter, minimum 5 µm in length, hovering above the substratum connecting two cells. TNTs were distinguished from cytoplasmic bridges, which appear following cell division, by the lack of a midbody clearly visible by DIC and/or staining of cellular membranes [59]. 8-well µ-slides (Ibidi) were pre-coated with fibronectin (10 µg/ml, F2006, Sigma-Aldrich) for 30 min at 37°C before washed with saline. About 20 000 cells were seeded per well and incubated overnight under physiological conditions. For TNT identification, plasma membranes were stained (8 min at 37°C) with 1.67 g/ml WGA–Alexa Fluor^©^ 594 or 488 in medium, followed by two gentle washes with saline or PBS always leaving 100 µl in the well. Cells were examined live by fluorescent light microscopy (Zeiss Axio Observer Z1 with AxioVision 4.8.2 or Zen software) using a 63x oil objective, heat block (37°C) and standard air conditions. One hundred cells per well were counted following a fixed counting pattern with 3-5 cells examined per vision field. Number of TNTs between cells were determined and expressed per 100 cells. The definition of a TNT in the present study combined with an outline of how they are counted is illustrated in Supplementary Figure 2D. In this example ten cells are shown where all structures are located above the substratum.

Two wells are counted for each condition in each experiment and experiments were performed three times or more, if not otherwise noted. Cell viability was monitored by Hoechst 33342 (Sigma) staining as described earlier [60].

### Immunofluorescence

TNT status of F-actin and microtubule was investigated (cells in 8-well μ-slides) in cells fixed (4% PFA in PBS, 0.2% glutaraldehyde in PBS) for 20 min at room temperature (RT) followed by one wash with PBS. Cells were incubated with WGA-Alexa 488 or 594 for 8 min followed by one wash with PBS, before permeabilized for 1 min (0.2% Tween^©^ in PBS), washed twice with PBS and blocked (0.5% Bovine Serum Albumin Fraction V (BSA) in PBS) for 20 min at RT. Cells were incubated for 1 h at RT in the dark with 33 nM AlexaFluor^©^ phalloidin, washed once with PBS and incubated with anti-β-tubulin antibody (1:200 in blocking solution) overnight at 4°C. Cells were washed twice with PBS and incubated with Alexa-488 or 594 goat-anti-mouse antibodies (1:5000 in blocking solution) for 1 h at RT, before washed twice with PBS and examined by fluorescence microscopy.

For subcellular localization studies, 50 000 cells were cytospun (Shandon cytofunnel, Thermo Scientific) onto coverslips (4 min, 400 rpm), fixed (4% PFA in PBS) for 20 min at RT before washed once with PBS and permeabilized with ice-cold methanol and incubated at -20°C for at least 20 min. Cells were blocked (0.5% BSA in PBS) for 15 min at RT before incubated overnight at 4°C with primary antibodies diluted in blocking buffer; TNFα-IP2 (1:100), RalA (1:200), LST1 (1:100), NF-кB-p65 (1:100), NF-кB-p-p65 (1:100). The cells were washed 3×5 min with PBS before incubated with Alexa-488 or 594-conjugated-secondary antibodies (1: 5000) diluted in blocking solution for 1 h at RT in the dark. The coverslips were washed 3×5 min with PBS before mounted on glass slides containing 5 µl VECTASHIELD mounting medium with DAPI (4,6-diamino-2-phenylindol-dihydrochlorid, Vector Laboratories) and examined by fluorescence microscopy.

### Scanning electron microscopy (SEM)

OCI-AML3 cells (500 000) were seeded onto L-lysine pre-coated coverslips followed by incubation at 37°C overnight before fixed (4% glutaraldehyde in 0.2 M Na-cacodylate in buffer diluted 1:1 with medium) for 2 h at RT. Cells were washed carefully three times for 15 min with 0.1 M Na-cacodylate buffer followed by a 60 min post-fixation with 1% osmiumtetraoxide in 0.1 M Na-cacodylate buffer and washed twice for 10 min with 0.1 M Na-cacodylate buffer. Dehydration with ethanol was performed with 30% for 15 min, 50% for 15 min, 70% for 20 min or overnight, 96% for 20 min and twice with 100% for 20 min. The coverslips were obtained from the wells and placed on SEM stubs before incubated in a heat-incubator overnight. Critical point drying was omitted in order to avoid breakage of TNTs. The SEM stubs were coated with 5-10 nm gold/palladium before SEM microscopy.

### Immunoblotting

Cells were lysed and analyzed by immunoblotting according to standard protocol [61, 62]. Developed immunoblots were detected, captured and quantified by ImageQuant LAS 4000 (GE Healthcare Life Sciences). Quantification of immunoblot bands were performed on 16-bit original files using 1D gel analysis option in ImageQuant TL version 8.1 (GE Healthcare). Lane creation was performed with the manual option and background was subtracted using the manual baseline option in this program. The final value after background subtraction was used for calculating the ratio relative to the loading control.

### Pull-down assay

The activity of Cdc42 was measured using an active Cdc42 pull-down and detection kit performed according to the manufacture's protocol (16119, Thermo Fisher Scientific). Briefly, 10-15×10^6^ cells were untreated or treated with 1 µM cytarabine for 1 h, followed by cellular lysis using the lyse/wash/binding buffer provided in the kit after addition of 1x EDTA free inhibitor cocktail (Thermo Scientific). The lysates were centrifuged for 15 min at 16 000 x g at 4°C. Protein contents were measured by the Bradford protein assay. 800 µg protein of each condition was used for the pull-down with glutathione resin and agarose beads together with 20 µg GST-PAK1-PBD. The GTPγS and GDP controls were performed according to manufature's protocol. About 30 µl of pull-down samples were applied onto an SDS-gel and Cdc42 was detected by immunoblotting using the anti-Cdc42 antibody provided in the kit.

### NF-κB-GFP-Jurkat reporter assay

NF-κB-GFP-Jurkat cells (500 000) were seeded in 24-well plates in a total volume of 1 ml. The negative control were the cells only and the positive control, cells added TNFα (5 ng/ml) for 24 h. The NF-κB-GFP-Jurkat cells were treated with cytarabine (1 μM) and daunorubicin (100 nM) separately and in combination without and with the addition of TNFα (5 ng/ml) for 24 h. Cells were collected, centrifuged and washed with PBS before 100 00 live cells were acquired per sample and analyzed by Guava easyCyte^TM^ (Merck Millipore) flow cytometer using Guava Soft version 2.2.2 software. Flow Cytometry results were analyzed using FlowJo 7.6 (Treestar). Results are representative of three independent experiments.

### Daunorubicin in SAOS-2 cells

SAOS-2 cells were seeded on a fibronectin coated 8-well µ-slide (IBIDI) one day before incubating for 2 h with 100 nM daunorubicin, followed by WGA-Alexa 488 staining and incubation in saline until analyzed by live cell microscopy.

### Co-culture

Cells were stained with pre-warmed cell tracker blue CMAC (7-amino-4-chloromethylcoumarin, 25 µM) diluted in medium without supplements for 30-60 min at 37°C, centrifuged and incubated for 30 min in pre-warmed complete medium before washed twice with saline. Cells were incubated overnight in medium with or without drugs. If treated, the cells were washed twice with saline before seeded onto 8-well μ-slides for co-culturing of cells.

### Lysotracker staining

Cells were incubated with 1 µM lysotracker green DND-26 for at least 30 min before washed once with saline and incubated with medium overnight or alternatively stained with WGA and investigated by fluorescent microscopy or by Zeiss LSM 510 confocal microscope with alpha Plan-Apochromat 63x/1.40 oil objective. Using following laser sets: Argon (Ar) - 30mW/488 and Helium/Neon I (HeNeI) - 1mW/543.

### Statistical analysis

Differences between two groups were analyzed by two-tailed unpaired t-test using GraphPad Prism 6 Version 6.03. An F-test was performed to verify that the internal variance in the groups were not significant. Significant difference was considered by a P-value < 0.05. All data with error bars are presented as mean ± S.D.

## SUPPLEMENTARY MATERIALS FIGURES AND MOVIES








