# Supplementary material for: Tunneling nanotube (TNT) formation is downregulated by cytarabine and NF-κB inhibition in acute myeloid leukemia (AML)
Source: Oncotarget. 2016 Dec 10;8(5):7946–63. doi: 10.18632/oncotarget.13853 (PMC5352373; doi:10.18632/oncotarget.13853)
Supplement: Supplementary file 1 [file oncotarget-08-7946-s001.pdf]

Tunneling nanotube (TNT) formation is downregulated by cytarabine and NF-κB inhibition in acute myeloid leukemia (AML)

SUPPLEMENTARY FIGURES AND MOVIES

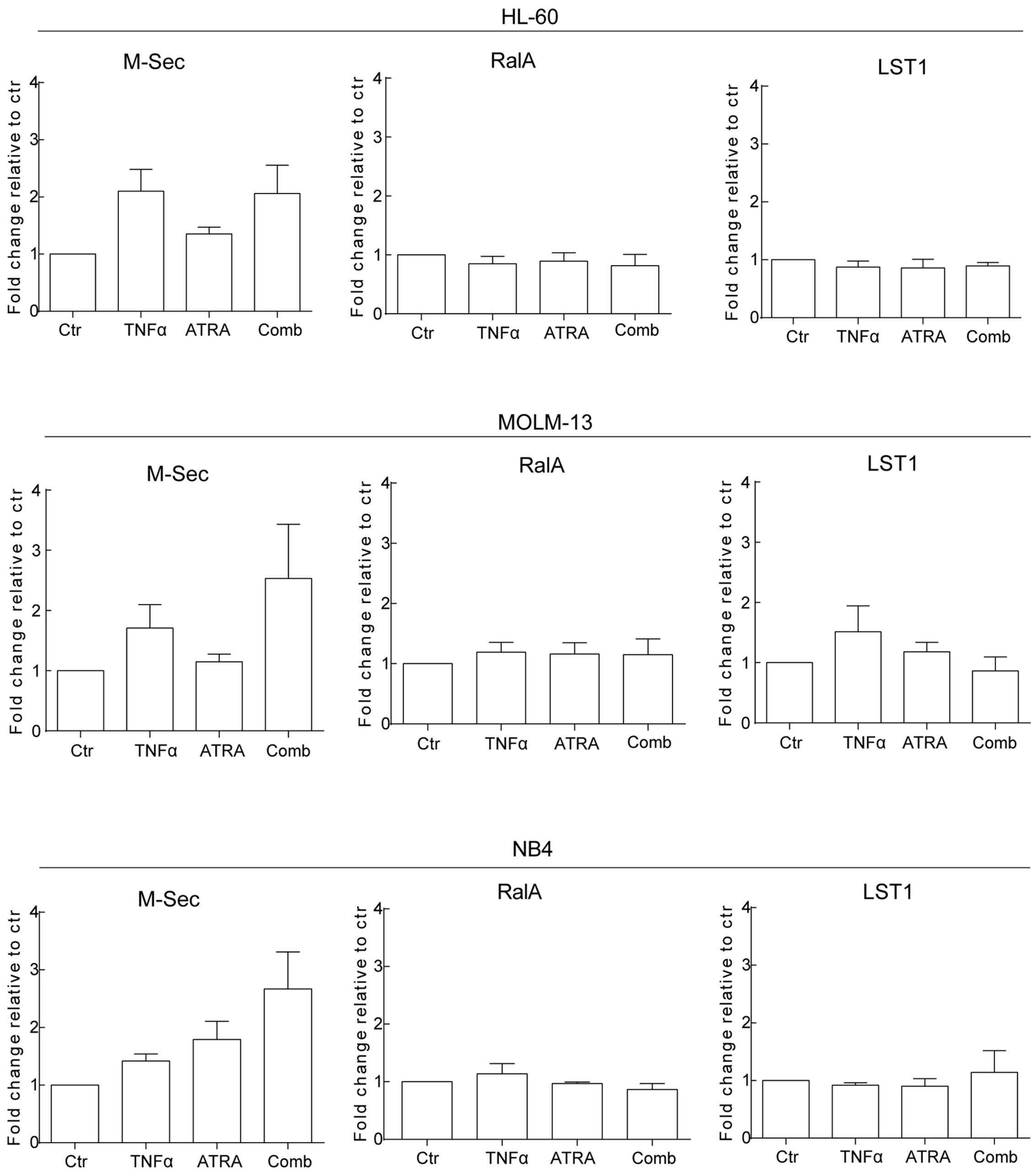

Supplementary Figure S1: M-Sec, RalA and LST1 expression in HL60, MOLM-13 and NB4 cells after TNFα and ATRA treatments. Immunoblot quantifications of experiments in Figure 3D. Data represent three independent experiments presented as mean ± S.D.

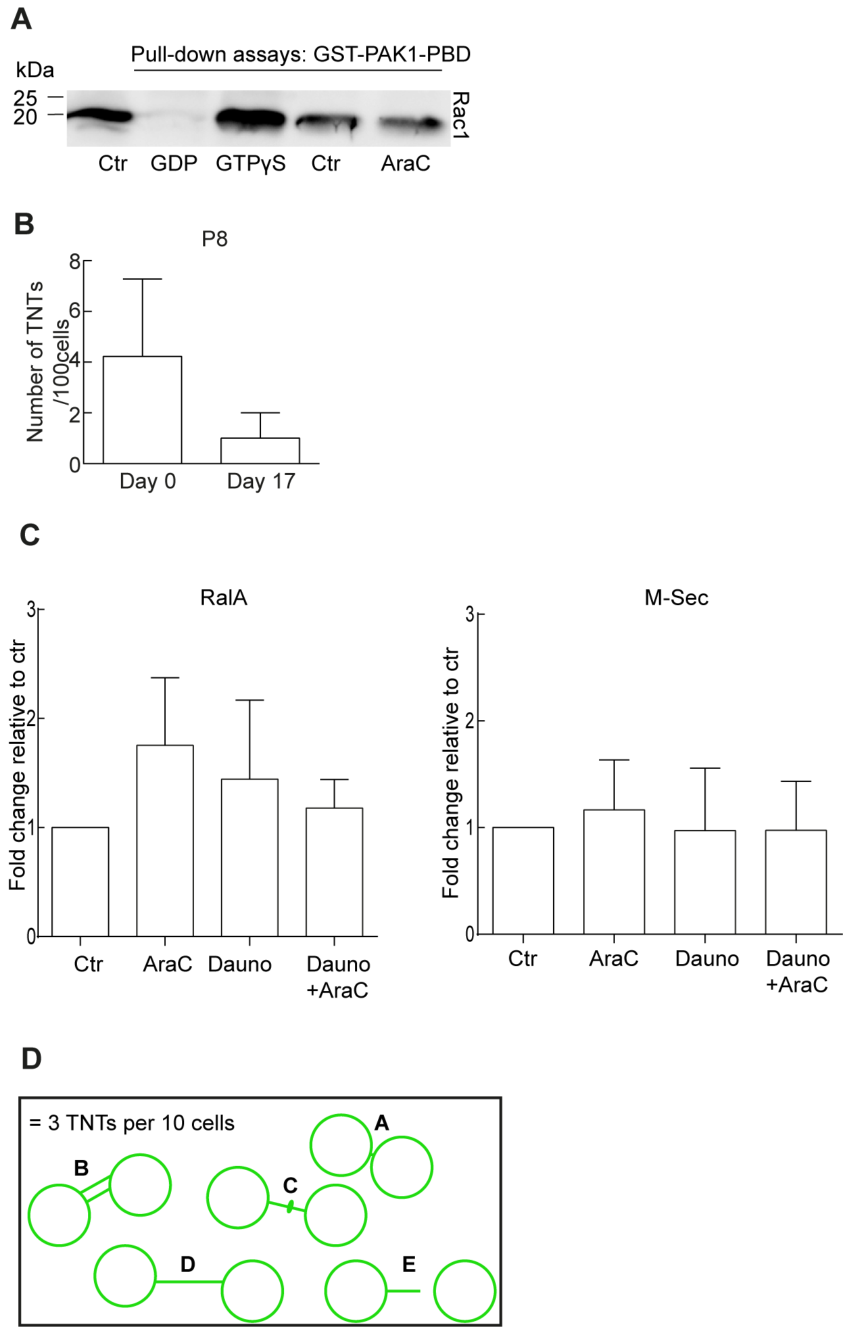

Supplementary Figure S2: A. Immunoblotting with anti-Rac1 antibody on pull-down with GST-PAK-PBD from experiment in Figure 6C **B.** TNT quantification in AML cells derived from the peripheral blood of an AML patient at diagnosis (day 0) and at day 17 after induction chemotherapy (AraC + daunorubicin). Experiment performed in duplicate. **C.** M-Sec and RalA expression in OCI-AML3 cells after cytarabine and daunorubicin treatments. Immunoblot quantifications of experiments in Figure 6E. Data represent three independent experiments presented as mean ± SD. **D.** The definition of a TNT in the present study combined with an outline of how they are counted. In this example ten cells are shown where all structures are located above the substratum. The following are not counted as TNTs; (A) a TNT formed between two cells that is < 5mm, (C) a cytoplasmic bridge after cell division containing a midbody and (E) a TNT not in contact with a recipient cell. The following are counted as TNTs; (D) one TNT above 5 mm interconnecting two cells, (B) two TNTs above 5 mm interconnecting two cells. In total 10 cells have been counted in this example and a total of 3 TNTs was registered per 10 cells.

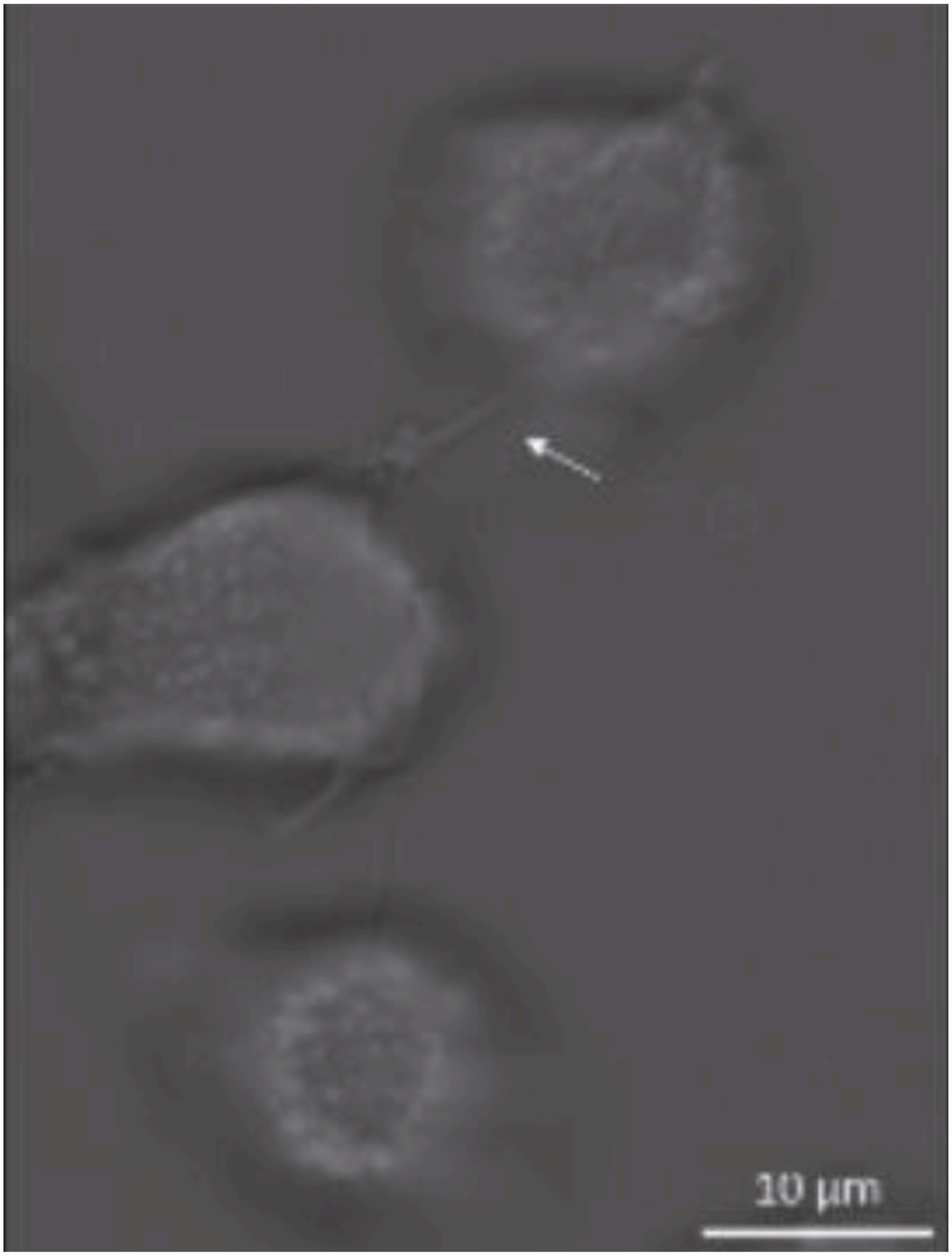

Supplementary Movie S1: Formation of TNTs from filopodia interplay in live OCI-AML3 cells. Images were taken every 30th sec using DIC microscopy. The arrow indicates formation of a TNT from filopodia interplay and a separate TNT is demolished. Scale bar = 10 µm.

See Supplementary Movie File 1

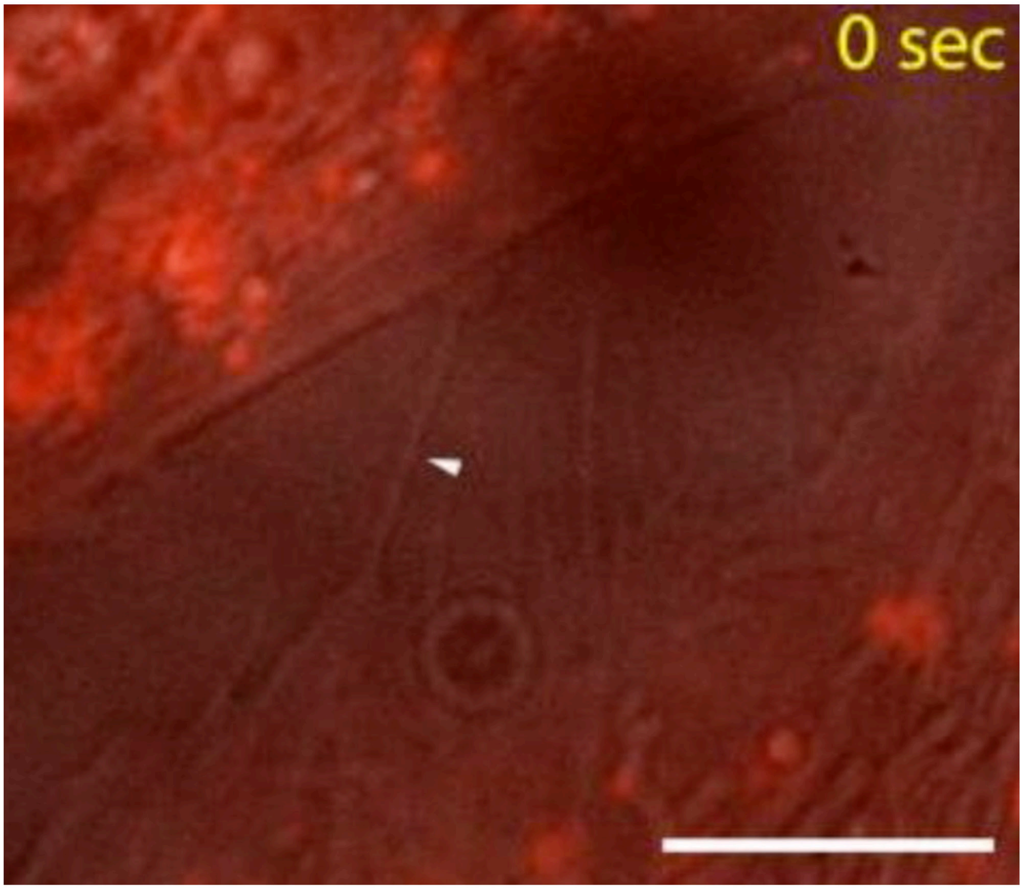

Supplementary Movie S2: SAOS-2 cells were incubated with daunorubicin for 2 h followed by live fluorescent and DIC microscopy where images were collected every 15th sec. Arrowheads indicate transport of daunorubicin in a TNT connecting two SAOS-2 cells, while arrows indicate no movement of daunorubicin not associated with the TNT during the same time frame. Scale bar = 10 µm.

See Supplementary Movie File 2

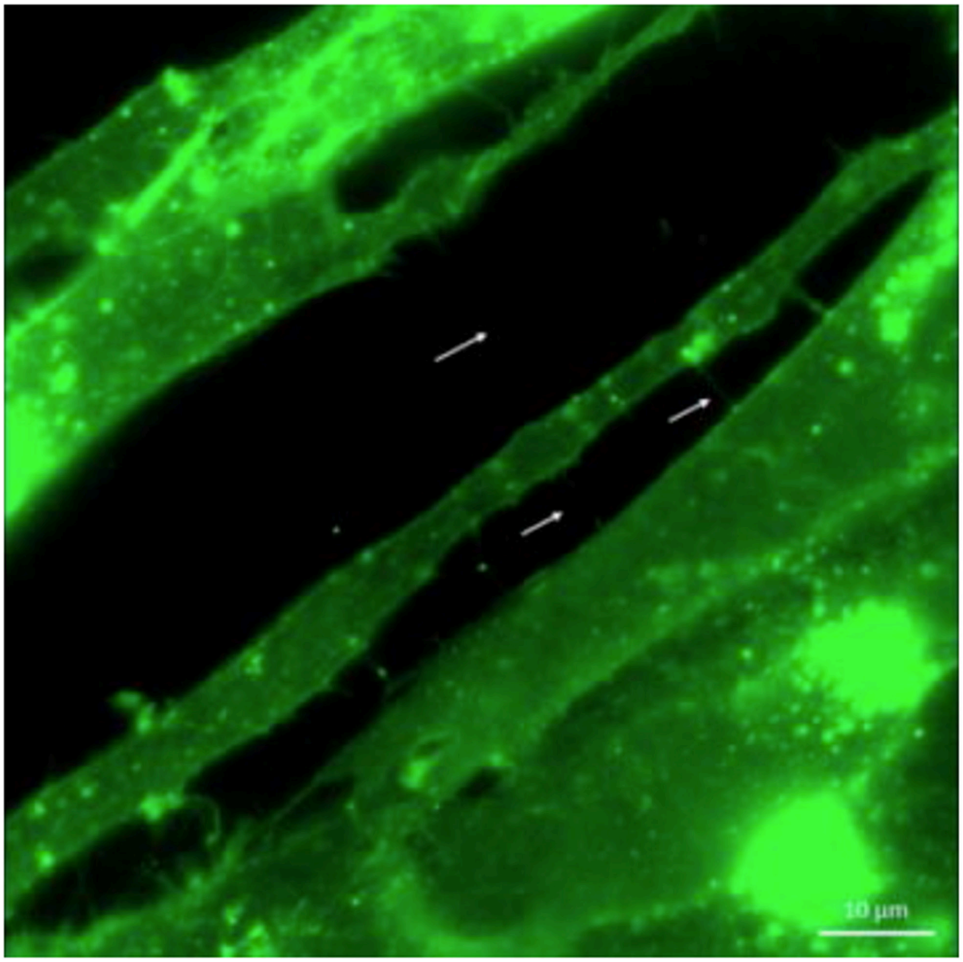

Supplementary Movie S3: SAOS-2 cells were stained with WGA-Alexa 488 and images were collected every 10th sec for a total of 180 seconds. Arrows indicate TNTs connecting SAOS-2 cells. Two shorter TNTs are sustained during the time-lapse while the long TNT is stretched and then breaks. Scale bar = 10 µm.

See Supplementary Movie File 3
